# Myocardial blood flow and myocardial flow reserve values in ^13^N–ammonia myocardial perfusion PET/CT using a time-efficient protocol in patients without coronary artery disease

**DOI:** 10.1186/s41824-018-0029-z

**Published:** 2018-06-11

**Authors:** Opstal TSJ, Knol RJJ, Cornel JH, Wondergem M, van der Zant FM

**Affiliations:** 1 Department of Nuclear Medicine, North West Clinics, Alkmaar, The Netherlands; 2 Cardiac Imaging Division Alkmaar, North West Clinics, Alkmaar, The Netherlands; 3 Department of Cardiology, North West Clinics, Wilhelminalaan 12, 1815 JD Alkmaar, The Netherlands

**Keywords:** Myocardial PET/CT, 13 N–ammonia, Stress flow, Flow reserve, Residual activity correction, Time-efficient protocol

## Abstract

**Background:**

Cardiac imaging by means of myocardial Positron Emission Tomography/Computed Tomography (PET/CT) is being used increasingly to assess coronary artery disease, to guide revascularization decisions with more accuracy, and it allows robust quantitative analysis of both regional myocardial blood flow (MBF) and myocardial flow reserve (MFR).

Recently, a more time-efficient protocol has been developed in combination with a residual activity correction algorithm in which a stress acquisition is performed directly after completion of the rest acquisition to subtract remaining myocardial radioactivity.

The objective of this study is to define flow values of myocardial blood flow (MBF) and Myocardial Flow Reserve (MFR) with ^13^N–ammonia (^13^NH_3_) myocardial perfusion PET/CT on patients without coronary artery disease using a time-efficient protocol, since reference values for this particular type of study are lacking in literature. In addition, we aim to determine the effect of the residual activity correction algorithm in this time-efficient protocol.

**Results:**

A mean MBF in rest of 1.02 ± 0.22 ml/g/min, a mean MBF in stress of 2.54 ± 0.41 ml/g/min with a mean MFR of 2.60 ± 0.61 were measured. Female patients had a significant higher MBF in rest and stress, but lower MFR; a small but significant negative correlation was measured between age and MBF in stress and MFR. Residual activity correction had a significant effect resulting in a difference in global stress MBF before and after correction of 0.39 ± 0.13 ml/g/min.

**Conclusions:**

This study established flow values for ^13^NH_3_ myocardial PET/CT with a time-efficient protocol, and established that MBF in stress corrected for residual activity is comparable with known reference values in normal studies without temporal overlap. Further validation of the technique could be of value, e.g. by comparison to standard imaging without temporal overlap, or validation against catheterization results.

**Electronic supplementary material:**

The online version of this article (10.1186/s41824-018-0029-z) contains supplementary material, which is available to authorized users.

## Background

Cardiac imaging by means of myocardial Positron Emission Tomography/Computed Tomography (PET/CT) is being used increasingly to assess coronary artery disease (CAD) and to guide revascularization decisions with more accuracy. Against conventional myocardial perfusion scintigraphy, this modality appears to be cost-effective, of comparable diagnostic performance and has strong prognostic power (Dorbala and Di Carli 2014; Juneau et al. 2016; Merhige et al. 2007). In addition, myocardial perfusion PET/CT allows robust quantitative analysis of both regional myocardial blood flow (MBF) during rest and pharmacologically induced stress and myocardial flow reserve (MFR) (Gould et al. 2013).

Nitrogen-13 ammonia (^13^NH_3_) is a common tracer used for myocardial perfusion PET/CTs with a half-live of 9.96 min (Anagnostopoulos et al. 2013). This half-live results in an obligatory time-consuming pause of 30 to 60 min (Juarez-Orozco et al. 2016; Kawaguchi et al. 2017) and subsequent repositioning of the patient between rest and stress scans, to avoid overlap of remaining, yet undecayed, myocardial radioactivity of the rest scan. Recently, a more time-efficient protocol has been developed in which a stress acquisition is performed directly after completion of the rest acquisition. This protocol is implemented in combination with a residual activity correction algorithm embedded in Syngo MBF software package (Siemens Healthcare, Knoxville, Tennessee, USA) to subtract remaining myocardial radioactivity.

The objective of the present study is to determine flow values of MBF and MFR in ^13^NH_3_ myocardial perfusion PET/CTs of a regular outpatient population to which a time-efficient protocol with a residual activity correction algorithm was applied, since reference values for this particular type of study are lacking in literature. In this report, we summarize data of a cohort study in patients without any signs of CAD on PET/CT or during follow-up. In addition, we aim to determine the effect of the residual activity correction algorithm on stress MBF in this time-efficient protocol.

## Methods

### Patient selection

In this cohort study all patients that were referred to ^13^NH_3_ myocardial PET/CT were prospectively recorded in a database from December 2013 until November 2016. All patients gave written informed consent for the use of their data for scientific purposes. Besides our standard time-efficient imaging protocol and clinical management no additional measurements or actions affecting the patient were performed. Therefore, approval of the local ethical committee for the present study was not necessary since the study does not fall within the scope of the Dutch Medical Research Involving Human Subjects Act (section 1.b WMO, 26th February 1998).

Patients with a clinical history of CAD or signs of CAD on previous imaging studies were excluded. Patients with abnormal ^13^NH_3_ myocardial PET/CT results in terms of visual ischemia or infarction were also excluded, as were patients who showed coronary calcifications on the attenuation correction CT of the myocardial perfusion PET/CT. Patients with incomplete or visually suboptimal PET/CTs (e.g. due to motion artefacts) were also excluded. Patients displaying stress MBF below 1.8 ml/gm/min were excluded, since this somewhat arbitrary threshold value is considered below expected lower limits in previous literature (Gould et al. 2013; Hutchins et al. 1990) and could be caused by coronary microvascular disease.

The remaining patients were followed up after the ^13^NH_3_ PET/CT study until December 2016 by a thorough screening of the electronic medical records of each patient. In these records all emergency room presentations, hospital admissions, cardiac events, and cardiac and non-cardiac procedures in our hospital were recorded. Any events occurring in other hospitals are most likely recorded by our cardiologists during follow-up as outpatient. Patients were excluded if, according to these records, they suffered a major adverse cardiovascular event (MACE), defined as sudden cardiac death, non-fatal out of hospital cardiac arrest, acute coronary syndrome or atherosclerotic ischemic stroke.

### Image acquisition and reconstruction parameters

All image data were acquired in list mode on a Siemens Biograph-16 TruePoint PET/CT (Siemens Healthcare, Knoxville, Tennessee, USA) with the TrueV option (enabled the axial field of view of 21.6 cm). This 3D system consists of a 16-slice CT and a PET scanner with four rings of lutetium oxyorthosilicate detectors.

After a topogram acquisition (110 kVp, 25 mAs) used for patient positioning, a CT transmission scan [130 kVp, 25 mAs (ref.), pitch 0.95] was acquired. Subsequently, patients were injected with 300 MBq of ^13^NH_3_ at rest. A 12-min rest imaging acquisition was started simultaneously with the start of the rest ^13^NH_3_ infusion (3 ml ^13^NH_3,_ rate 0.4 ml/s followed with 17 ml NaCl and flushed with 20 ml NaCl at rate 2 ml/s). Pharmacologic stress was induced by an intravenous adenosine infusion (0.14 mg/kg/min for 6 min) immediately after acquisition of rest images. One minute after the start of the adenosine infusion, a 12-min stress imaging acquisition was started. Two minutes later, 400 MBq of ^13^NH_3_ was administered (3 ml, rate 0.4 ml/s). Immediately after completion of stress imaging, a second CT transmission scan was performed [130 kVp, 25 mAs (ref.), pitch 0.95].

Static, dynamic, and 16-bin ECG-gated images were generated from the list mode data. The acquired emission data were reconstructed using 3D attenuation-weighted ordered subsets expectation maximization (OSEM3D) reconstruction with a 168 × 168 matrix, zoom 2, a Gaussian filter with a full-width at half-maximum of 5 mm, two iterations, and 21 subsets for gated and dynamic images and TrueX (OSEM3D with PSF) reconstruction with a 256 × 256 matrix, zoom 2, a Gaussian filter of 4 mm, four iterations, and eight subsets for static images. CT-based attenuation, scatter, decay, and random corrections were applied to the reconstructed images.

Total scan duration was 25 min: 12 min for rest and 10.5 min for stress scans, with 2.5 min delay between these two parts. From each of the acquisitions dynamic, static and gated images were reconstructed. Dynamic rest images were reconstructed from the part of the dataset that was acquired directly after the initiation of the first ^13^NH_3_ injection, using 25 frames: 1 × 10, 12 × 5, 2 × 10, 7 × 30, 2 × 60, 1 × 180 s. MBF was subsequently computed by applying the Hutchins model to the dynamic images (Hutchins et al. 1990). For reconstruction of the gated and static rest images, the first 2.5 min of the rest acquisition data were skipped to allow for blood pool clearance and the subsequent 9.5 min of data were used. Gated images were used to assess left ventricular ejection fraction and regional wall motion abnormalities, while static images provide visualization of myocardial perfusion. The dynamic stress images were reconstructed from the part of the dataset that was acquired from 30 s before initiation of the second ^13^NH_3_ injection (which took place at exactly 15 min after the beginning of each scan) and onwards, using 26 frames for stress: 1 × 30, 1 × 10, 12 × 5, 2 × 10, 7 × 30, 2 × 60, 1 × 180 s. For reconstruction of static and gated stress images, the last 7.5 min of the acquisition data were used.

A cardiac specific motion correction algorithm from Siemens Molecular Imaging was used to detect the motion of the myocardium between the dynamic frames automatically. The motion was detected by a rigid image registration. The frames were re-aligned using an automatic motion correction method, propagating backwards from the final frame, comprising registrations between consecutive frames, until the frames no longer contained data that could be reliably registered due to change in image appearance as the sequence evolves.

Residual activity correction was applied for dynamic and static images obtained from the stress scan to exclude the interference of residual ^13^NH_3_ activity from the rest acquisition. Dynamic data sets were processed by subtraction of residual activity present in the first frame of the stress study (acquired directly before the stress ^13^NH_3_ injection) from the time activity curves using a residual correction method that was integrated into the Syngo MBF software package (Siemens Healthcare, Knoxville, Tennessee, USA). To assess the effect of this correction method, stress MBF values with and without residual activity correction will be provided. Static images were corrected by subtracting the residual activity measured at an interval of 2 min before the stress ^13^NH_3_ injection and corrected for decay and patient motion.

The quality of the registration between PET and CT was reviewed by experienced technologists and, in case of misalignment, corrected manually by means of 3D translations on the registration matrix before the final reconstruction process was started (Kan et al. 2016). MBFs were measured globally and for separate coronary artery territories and corresponding MFRs were calculated. Vital parameters were continuously monitored and rate-pressure products (RPP) calculated.

### Statistics

Statistical analysis was performed using SPSS software (version 20.0.0; IBM SPSS, Chicago, Illinois, USA). Categorical variables are presented as frequencies with percentages and continuous variables as mean ± standard deviation. Follow-up duration was described with median and interquartile range to better understand the distribution. Normal distribution was tested by the Shapiro-Wilk test. Data was subsequently divided by age and sex. Since most data was not normally distributed, significance between flow before and after residual activity correction was tested with the Wilcoxon Signed Ranks Test, significance of difference by sex with the Mann-Whitney U test, and significance of correlation by age with the 2-tailed Spearman’s rho test.

## Results

One thousand eight hundred forty-four patients were referred for ^13^NH_3_ myocardial PET/CT. After exclusion due to known CAD (*n* = 599), abnormal/positive ^13^NH_3_ PET/CT results (*n* = 879), coronary calcifications on CT scan (*n* = 1406), and/or incomplete or suboptimal scans (*n* = 48), 225 patients were found suitable to enter the study. 19 ^13^NH_3_ PET/CTs of these patients yielded stress MBFs below 1.8 ml/g/min, and those were also excluded. This resulted in 206 eligible patients. The average age of the resulting study group was 62 ± 11.7 years and 63 (31%) of the included patients were male. Baseline characteristics are listed in Table 1. During a median follow-up of 18 months (interquartile range 9–27) no MACEs were recorded in this group.

**Table 1:** Patient characteristics

Baseline characteristics	
Subjects	206
Male no	63 (31%)
Age, years	62 ± 12
BMI	27 ± 5
Follow-up, months^a^	18 (IQR = 9–27)
Smoker	29 (14%)
Diabetes	33 (12%)
Positive family history	70 (34%)
Hypercholesterolemia	61 (30%)
Hypertension	91 (44%)
MACE	0 (0%)

Values are n, mean ± standard deviation, or n (%)

*BMI* Body Mass Index. *MACE* Major Adverse Cardiac Event

^a^median and interquartile range (IQR)

**Fig. 1:**
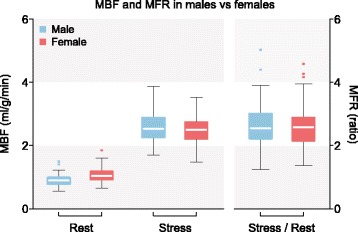
MBF and MFR by sex. Boxplot with Tukey whiskers, dots are outliers. MBF = Myocardial Blood Flow, MFR = Myocardial Flow Reserve

**Fig. 2:**
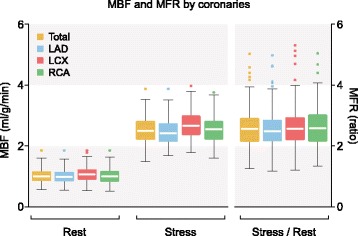
MBF and MFR divided by coronaries. Boxplot with Tukey whiskers, dots are outliers. MBF = Myocardial Blood Flow, MFR = Myocardial Flow Reserve. LAD = Left Anterior Descending, LCX = Left Circumflex, RCA = Right Coronary Artery

**Fig. 3:**
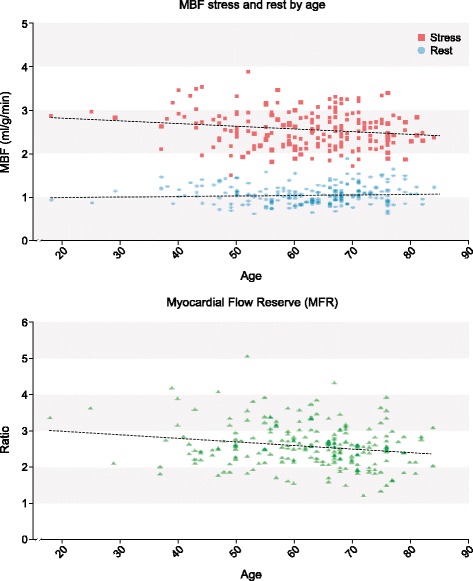
Scatter plot MBF stress/rest and MFR by age with linear regression analysis. Significant negative correlation of MBF in stress (*r* = − 0.139, *p* = 0.047) and MFR (*r* = − 0.165, *p* = 0.018) with age. MBF = Myocardial Blood Flow, MFR = Myocardial Flow Reserve

RPP, global rest MBFs, stress MBFs and resulting MFRs are listed in Table 2, further categorized by sex and shown as boxplots in Figs. 1 and 2. The corresponding complete dataset can be found in Additional file 1. The global rest MBF for all patients was 1.02 ± 0.22 ml/g/min and global stress MBF was 2.54 ± 0.41 ml/g/min which resulted in a global MFR of 2.58 ± 0.60. RPP was significantly higher in females compared to males in rest (9370 ± 2487 vs. 8149 ± 1490, *p* = 0.001) and stress (12,939 ± 2871 vs. 11,956 ± 2321*, p =* 0.001*).* Global rest and stress MBF were significantly higher in females compared to males as well, with a significantly lower global MFR in females (*p =* ≤ 0.012). A division by age is listed in Table 3 and scatter-plotted in Fig. 3.

Spearman’s rho test showed a minor but significant negative correlation of MBF in stress (*r =* − 0.139*, p* = 0.047*)* and MFR (*r* = − 0.165, *p* = 0.018) with age, while MBF in rest was not correlated with age. The effect of the residual activity correction is assessed in Table 4, by providing stress MBF with and without residual activity correction. The residual activity correction method significantly reduced the stress MBF values (*p* = < 0.001); a mean global MBF without residual activity correction was measured of 2.93 ± 0.44 ml/g/min with a calculated mean residual activity of 0.39 ± 0.13 ml/g/min, resulting in a mean global MBF with residual activity correction of 2.54 ± 0.41 ml/g/min.

**Table 2:** Mean Rate Pressure Product, Myocardial Blood Flow and Myocardial Flow Reserve with residual activity correction divided by sex

	Total (*n* = 206)	Male (*n* = 63)	Female (*n* = 143)	*P*-value (male/female)
RPP rest	8991 ± 2293^a^	8149 ± 1490^a^	9370 ± 2487^a^	0.001
RPP stress	12,939 ± 2871^a^	11,956 ± 2321	13,382 ± 2990^a^	0.001
MBF rest (ml/g/min)	1.02 ± 0.22^a^	0.90 ± 0.19	1.08 ± 0.22^a^	< 0.001
MBF stress (ml/g/min)	2.54 ± 0.41	2.43 ± 0.35	2.59 ± 0.43	0.012
MFR (stress/rest ratio)	2.60 ± 0.61^a^	2.81 ± 0.58^a^	2.50 ± 0.60^a^	< 0.001

Mean results ± standard deviation

*MBF* Myocardial Blood Flow, *MFR* Myocardial Flow Reserve, *RPP* Rate Pressure Product

^a^not normally distributed by Shapiro-Wilk test. Significance tested by Mann-Whitney U

**Table 3:** Mean Myocardial Blood Flow and Myocardial Flow Reserve with residual activity correction divided by age categories

Subgroup (*n*)	MBF rest (ml/g/min)	MBF stress (ml/g/min)	MFR (stress/rest ratio)
Total (206)	1.02 ± 0.22^a^	2.54 ± 0.41	2.60 ± 0.61^a^
Age < 50 (30)	1.05 ± 0.23	2.68 ± 0.48	2.66 ± 0.67
Age 50–59 (45)	0.99 ± 0.21	2.57 ± 0.43	2.73 ± 0.69^a^
Age 60–69 (73)	0.99 ± 0.22^a^	2.53 ± 0.39	2.66 ± 0.54
Age ≥ 70 (58)	1.08 ± 0.23	2.46 ± 0.36	2.39 ± 0.56^a^

Mean results ± standard deviation

*MBF* Myocardial Blood Flow, *MFR* Myocardial Flow Reserve

^a^not normally distributed by Shapiro-Wilk test

**Table 4:** Mean Myocardial Blood Flow before and after residual activity correction divided by territory

Territory	MBF stress before RAC (ml/g/min)	MBF stress after RAC (ml/g/min)	*P*-value
LAD	2.87 ± 0.45^a^	2.47 ± 0.41^a^	< 0.001
LCX	3.07 ± 0.45	2.69 ± 0.42	< 0.001
RCA	2.94 ± 0.47	2.55 ± 0.45	< 0.001
Global	2.93 ± 0.44	2.54 ± 0.41	< 0.001

Mean results ± standard deviation

*MBF* Myocardial Blood Flow, *RAC* Residual Activity Correction, *LAD* Left Anterior Descending, *LCX* Left Circumflex, *RCA* Right Coronary Artery

^a^not normally distributed by Shapiro-Wilk test

## Discussion

In this cohort study myocardial blood flow values were established for global rest and stress MBF and global MFR for time-efficient ^13^NH_3_ myocardial PET/CT with residual activity correction in patients without a history of CAD from a regular cohort of cardiac (out) patients who showed no signs of ischemia on the performed ^13^NH_3_ myocardial PET/CT and no coronary calcifications on CT. This resulted in a patient population with an excellent prognosis, since no major adverse cardiac events were recorded during follow-up.

An earlier review of Gould et al. summarized global values of rest MBF (0.79 ± 0.06 ml/g/min), stress MBF (2.66 ± 0.90 ml/g/min) and MFR (3.42 ± 1.02) of 859 normal controls who underwent a ^13^NH_3_ myocardial perfusion PET/CT (Gould et al. 2013). In these studies, a more common protocol was used with a delay of between rest and stress scans, typically 30 to 60 min, which negates the overlap between rest and stress images. Our study differs from most by applying a time-efficient acquisition and image processing protocol, which corrects for this overlap with a residual activity correction algorithm.

The rest MBF in the present study was higher (1.02 ± 0.22 vs. 0.79 ± 0.06 ml/g/min) compared to the data summarized by Gould et al. (Gould et al. 2013). However, the mean rest MBFs differ between the 64 individual studies included in the review, with a lowest mean rest MBF of 0.57 ± 0.15 ml/g/min and the highest mean rest MBF of 1.09 ± 0.20 ml/g/min. A closer look at the publications in this review shows significant differences in used method, software and hardware, which could explain the found range of mean rest MBF. While our rest MBF is on the higher end, it’s still within this range. The difference could not be explained by our time-efficient protocol, since rest MBF is not influenced by this method.

Residual activity had a significant effect on all stress MBFs, globally as well as in the separate territories, with a calculated global mean residual activity of 0.39 ± 0.13 ml/g/min. Notably, even though a time-efficient protocol was used with overlap, resulting in residual activity during the stress scans, the corrected stress MBF was similar compared to the data summarized by Gould et al. (2.54 ± 0.41 vs. 2.66 ± 0.90 ml/g/min). This resulted in lower MFR (2.60 ± 0.61 vs. 3.42 ± 1.02 ml/g/min, Tables 2 and 3) (Gould et al. 2013). A higher global rest and stress MBF with lower MFR was found in female patients. One explanation could be that female patients are more susceptible to mental stress before procedure (Marques et al. 2016), which would explain the increased RPP compared to males, resulting in increased MBF. Age had a small but significant negative correlation with global stress MBF and MFR, but previous studies have been contradictory on this matter (Bernacki et al. 2016; Czernin et al. 1993). The decline in the present study may be explained by a progression of coronary microvascular disease with age (Marinescu et al. 2015), by an increase of risk factors by age, or by currently unknown factors.

An alternative method to determine reference values could be obtained by performing ^13^NH_3_ myocardial PET/CT scans with a time-efficient protocol on healthy patients with no risk factors. However, since in these low risk patients no additional testing or coronary computed tomography angiography is preferred to stress imaging (Task Force Members et al. 2013), these patients would not be representative to the average cardiac (outpatient) clinic population.

Our study adds to existing literature by establishing myocardial blood flow values on ^13^NH_3_ myocardial PET/CT scans using a time-efficient protocol of patients with no sign of CAD, offering stress MBF values which agree with known reference values in normal studies without a time-efficient protocol. In addition, to our knowledge this is the first study to present the effects of temporal overlap and the subsequent obligatory residual activity correction method in a large number of patients. The time-efficient acquisition protocol allows for shorter test duration, since rest and stress components are performed in short order without having to reposition the patient. The data sets in this study were processed by the Syngo MBF software package. While comparison studies between different software packages are limited, a recent study demonstrates a moderate to good agreement in myocardial perfusion quantification between Syngo MBF and other software packages (Nesterov et al. 2017).

Residual activity correction is a relatively novel technique. To our knowledge, the application of ^13^NH_3_ myocardial PET/CT scans using a time-efficient protocol with residual activity correction has been described in only two small studies. One study compares difference in MBF with and without residual activity correction of the same measurements, which were significant in the LAD and RCA territory, and concludes that the overestimation of stress MBF can be avoided by applying residual activity correction (Markousis-Mavrogenis et al. 2017). The other study comprises a preliminary validation of residual activity correction in a pilot study comparing two rest-acquisitions in 31 subjects, one with temporal overlap and residual activity correction and one without temporal overlap, and confirms matching myocardial blood flow values and statistical distribution (Lazarenko et al. 2014). While these findings are interesting, little detail is provided, and the number of subjects is small. Further validation of the technique could be of value, for example by direct comparison to standard imaging without temporal overlap, or validation against catheterization results.

## Conclusions

Establishment of myocardial flow values for ^13^NH_3_ myocardial PET/CT with a time-efficient protocol using residual activity correction yielded a global rest MBF of 1.02 ± 0.22 ml/g/min global stress MBF of 2.54 ± 0.41 ml/g/min and a resulting global MFR of 2.60 ± 0.61 for patients without CAD. Female patients showed a significantly higher MBF in both rest and stress compared to men, and age had a small but significant negative correlation with average stress MBF and MFR. Residual activity correction had a distinct significant effect resulting in a difference in global mean stress MBF before and after correction of 0.39 ± 0.13 ml/g/min.

Residual activity correction provides a time-efficient protocol, which results in reliable MBF value measurements in line with previous literature. Future validation for this novel method should focus on a direct comparison to standard imaging without temporal overlap and to catheterization results.

## Additional file

Additional file 1: Complete dataset in .sav format. (SAV 88 kb)

## Abbreviations

^13^NH_3_: Nitrogen-13 ammonia
CAD: Coronary artery disease
FFR: Fractional flow reserve
MACE: Major adverse cardiovascular event
MBF: Myocardial blood flow
MFR: Myocardial flow reserve
PET/CT: Positron Emission Tomography/Computed Tomography
RPP: Rate pressure product
